# The N6-Methylandenosine-Related Gene *BIRC5* as a Prognostic Biomarker Correlated With Cell Migration and Immune Cell Infiltrates in Low Grade Glioma

**DOI:** 10.3389/fmolb.2022.773662

**Published:** 2022-03-03

**Authors:** Xiulin Jiang, Yulin Shi, Xi Chen, Haitao Xu, Xiaobin Huang, Lihua Li, Jun Pu

**Affiliations:** ^1^ Department of Neurosurgery, the Second Affiliated Hospital of Kunming Medical University, Kunming, China; ^2^ Key Laboratory of Animal Models and Human Disease Mechanisms of Chinese Academy of Sciences and Yunnan Province, Kunming Institute of Zoology, Kunming, China; ^3^ Kunming College of Life Science, University of Chinese Academy of Sciences, Beijing, China; ^4^ Kunming Medical University, Kunming, China

**Keywords:** LGG, BIRC5, M6A RNA methylation, cell migration, immune infiltration, drug sensitivity

## Abstract

Gliomas account for 75% of all primary malignant brain tumors in adults and are associated with high mortality. Emerging evidence has demonstrated that baculoviral inhibitor of apoptosis repeat containing 5 (*BIRC5*) plays a critical role in cell apoptosis and the progression of diverse cancers. However, no studies have yet focused on the immunological function and mechanisms of upstream *BIRC5* regulation in the progression of low-grade gliomas (LGG). Here, we evaluated *BIRC5* expression and clinical characteristics in people with LGG using the Chinese Glioma Genome Atlas, The Cancer Genome Atlas, Gene Expression Omnibus, Rembrandt, and Gravendeel databases. We used Kaplan–Meier statistics and receiver operating characteristic (ROC) curves to analyze the prognostic value of *BIRC5* in LGG. Kyoto Encyclopedia of Genes and Genomes (KEGG) and Gene Ontology (GO) enrichment terms were also explored to identify functional roles of *BIRC5*. The Tumor Immune Estimation Resource (TIMER) and Tumor Immune System Interaction (TISIDB) databases were used to examine the correlation between *BIRC5* expression and immune cell infiltration in LGG. The Genomics of Drug Sensitivity in Cancer (GDSC) and Cancer Therapeutics Response Portal (CTRP) databases were used to examine the potential drugs targeting *BIRC5*. We used transwell and wound healing assays to determine the biological functions of *BIRC5* in glioma cell migration. Our results demonstrated that *BIRC5* was highly expressed in LGG and the expression level correlated with tumor grade, prognosis, histological subtype, isocitrate dehydrogenase 1 (*IDH1*) mutation, 1p/19q chromosomal co-deletion, chemotherapy status, and O[6]-methylguanine-DNA methyltransferase (MGMT) promoter methylation status. GO and KEGG analysis showed that *BIRC5* is primarily involved in cell proliferation and immune response-related signaling pathways. We also found that *BIRC5* was significantly correlated with m6A modification and diverse drug sensitivity. TIMER and TISIDB database analysis showed that *BIRC5* expression is associated with infiltration of diverse immune cells and immune modulation in LGG. *BIRC5* knockdown inhibited LGG cell migration. Collectively, our results demonstrate that *BIRC5* is correlated with cell migration and immune infiltration in LGG and may be a useful prognostic biomarker.

## 1 Introduction

Low-Grade Glioma (LGG) is a relatively common tumor in the central nervous system that mainly includes World Health Organization (WHO) grade 2 and 3 gliomas (Ostrom et al., 2014). Mounting evidence has demonstrated that the molecular characteristics of gliomas include mutated isocitrate dehydrogenase 1 and 2 genes (IDH1/2) and co-deletion of 1p/19q (Aldape et al., 2019). Despite surgical advances and progress in adjuvant therapeutic strategies, clinical outcomes in people with LGG are still poor. Therefore, uncovering the molecular mechanisms underlying the initiation and progression of LGG and identifying highly reliable biomarkers is crucial to improving the diagnosis and treatment of people with this cancer type.

Members of the *BIRC* gene family (*BIRC1–8*) mainly encode inhibitors of apoptosis (LaCasse et al., 1998; Makuch-Kocka et al., 2021). These genes are reported to play important roles in cell cycle regulation, immune system activities, and signal transduction (Liang et al., 2020). Aberrant expression of these genes also contributes to the progression of diverse cancers: for instance, OCT4 elevates the promoter activity of *BIRC5*, contributing to the progression of hepatocellular carcinoma (Cao et al., 2013). It has been shown that *BIRC5* is highly expressed in breast cancer, and its expression is correlated with relapse-free survival and overall survival; it, therefore, may have potential as a useful predictive marker and therapeutic target in this context (Dai et al., 2020). Furthermore, a recent study indicated that microRNA-203 inhibits the expression of *BIRC5* and suppresses the proliferation and migration of human triple-negative breast cancer cells (Wang et al., 2012). Multiple studies have found that *BIRC* genes have a pivotal role in multiple physiological processes including cell growth, cell cycle regulation, immune responses, and cancer progression (Pu et al., 2020). However, no studies have yet focused on the immunological function and upstream regulatory mechanism of *BIRC* genes in the progression of LGG: in this study, we therefore aimed to investigate the role of *BIRC* genes in these processes.

By integrated analysis of the genomic, transcriptomic, and clinical data from the Chinese Glioma Genome Atlas (CGGA), The Cancer Genome Atlas (TCGA), Gene Expression Omnibus (GEO), Rembrandt and Gravendeel datasets, we found that the RNA and protein of *BIRC5* were significantly up-regulation in LGG tissue, high level of *BIRC5* was correlated with tumor grade and poor prognosis in LGG. Additionally, *BIRC5* expression is also associated with histology subtypes, IDH1 mutation, 1p/19q chromosomal co-deletion, chemotherapy status, and MGMT promoter methylation status, well-established molecular characteristics of gliomas. The potential signaling pathway participated by *BIRC5* in LGG was examined by GSEA. Furthermore, our results demonstrated that *BIRC5* expression in LGG was regulated by DNA hypo-methylation, the correlation between *BIRC5* and immune cell infiltration was evaluated. Finally, qRT-PCR was used to validate the expression of *BIRC5* in normal human astrocytes cells and glioma cell lines. Knock-down of *BIRC5* significantly inhibits the cell migration of gliomas cells *in vitro*. The findings in our study demonstrated that the crucial role of *BIRC5* in LGG patients, and discovered an underlying mechanism between *BIRC5* and immune response.

## 2 Materials and Methods

### 2.1 Gene Expression and Survival Analysis

We download the RNA expression and clinical data of glioma cohort projects from The Cancer Genome Atlas (TCGA) research program (https://www.cancer.gov/tcga), including 505 cases of lower-grade glioma (LGG) and 155 cases of GBM RNA sequencing counts data with clinical information. The various normal tissues expression data obtained from the Genotype-Tissue Expression Project (GTEx) RNA sequencing data as resources (https://www.genome.gov/Funded-Programs-Projects/Genotype-Tissue-Expression-Project). This dataset was utilized analysis the expression of *BIRC5* in glioma and used to examine the correlation between *BIRC5* and various clinical features. We also download the glioma RNA-seq dataset “mRNAseq_693” recruited in the Chinese Glioma Genome Atlas (CGGA) (HTTP: //www.cgga.org.cn/analyse/RNA-data.) contains 693 glioma samples were utilized as verification of the expression and prognosis of *BIRC5* in glioma. The GSE4290, GSE16011 andGSE50161 dataset was downloaded from The Gene Expression Omnibus (GEO, http://www.ncbi.nlm.nih.gov/geo/) database, used to analyze the expression of *BIRC5* in low-grade glioma.

### 2.2 GEPIA Analysis

The GEPIA website (http://gepia.cancer-pku.cn/) was used to explore prognosis of *BIRC* family gene in LGG. Furthermore, we used GEPIA database examine the correlation between *BIRC5 expression* and pathological stage of various human cancers.

### 2.3 Univariate and Multivariate Cox Analyses

Univariate and multivariate Cox analyses were conducted by using the R package “survival.” The WHO grade, IDH status, 1p/19q codeletion, primary therapy outcome, gender, age, and *BIRC5* expression were included in these analyses. The receiver operating characteristic (ROC) analysis was performed with the R package “pROC.”

### 2.4 Gene Set Enrichment Analysis and Kyoto Encyclopedia of Genes and Genome Analyses

Gene expression data of LGG in HTSeq-Counts were downloaded from TCGA website for further analysis. The correlated genes of *BIRC5* were screened with Pearson’s correlation coefficients (|r| >0.6 and *p* < 0.001) with R package “deseq2.” According to the default statistical methods, an adjusted *p*-value < 0.05 was considered significant. GO and KEGG analysis were conducted on *BIRC5* correlated genes with R package “clusterProfiler” to identify the possible biological functions and signaling pathways affected by *BIRC5*. In the present research, the gene set kegg.v6.2.symbols.gmt, which served as a reference gene set, was downloaded from the Molecular Signatures Database (MSigDB) (http://software.broadinstitute.org/gsea/msigdb). We performed GSEA to examine the potential signaling pathway participated by *BIRC5* in low-grade glioma.

### 2.5 Tumor Immune Estimation Resource

TIDIDB (http://cis.hku.hk/TISIDB/), an integrated repository portal for tumor-immune system interactions, utilized to analysis of the expression of *BIRC5* in diverse immune subtype of low-grade glioma (Ru et al., 2019). TIMER (https://cistrome.shinyapps.io/timer/) (Li et al., 2017), an interactive web portal, could perform a comprehensive analysis on the infiltration levels of different immune cells. In this study, we used the TIMER database to explore the correlation between *BIRC5* and diverse immune cell infiltration, and different immune-related gene marker in low-grdae glioma.

### 2.6 DNA Methylation Analysis for BIRC5

We utilized MethSurv (https://biit.cs.ut.ee/methsurv/), a web tool to perform multivariable survival analysis using DNA methylation data, to analyze the diverse DNA methylation sites in the promoter of *BIRC5* in glioma (Modhukur et al., 2018). Furthermore, the SMART (http://www.bioinfo-zs.com/smartapp/), an interactive web application for comprehensive DNA methylation analysis and visualization, was used to examine the correlation between *BIRC5* expression and DNA methylation in glioma (Li et al., 2019).

### 2.7 Analysis of the Correlation Between the *BIRC5* Expression and Drug Sensitivity

We utilized the GDSC (https://www.cancerrxgene.org/) and CTRP (http://portals.broadinstitute.org/ctrp.v2.1/) databases to analyze the correlation between *BIRC5* expression and drug sensitivity (Basu et al., 2013; Yang et al., 2013).

### 2.8 Cell Culture, RNA Isolation, and Real-Time PCR

The gliomas cell lines were purchased from the cell bank of Kunming Institute of Zoology, and cultured in DMEM medium (Corning) supplemented with 10% fetal bovine serum (FBS) and 1% penicillin/streptomycin. HEK-293T cells were cultured in DMEM medium (Corning). Total RNA was extracted according to the manufacturer’s protocol, and then reverse-transcribed using RT reagent Kit (Takara Bio, Beijing, China, Cat# RR047A; TIANGEN Biotech, Beijing, China, Cat# KR211-02). Real-time PCR was performed by FastStart Universal SYBR Green Master Mix (Roche, Cat# 04194194001; TIANGEN Biotech, Beijing, China, Cat# FP411-02) using an Applied Biosystems 7500 machine. The primers used in this study are shown in the following, *BIRC5*-F: AGG​ACC​ACC​GCA​TCT​CTA​CAT, *BIRC5*-R: AAG​TCT​GGC​TCG​TTC​TCA​GTG; β-actin-F: CTTCGCGGGCGACGAT, β-actin-R: CCA​TAG​GAA​TCC​TTC​TGA​CC. The expression quantification was obtained with the 2^−ΔΔCt^ method.

### 2.9 Plasmid Construction and siRNA Interference

The *BIRC5* siRNA, control siRNA were synthesized by GenePharma (Shanghai, China), and a scrambled siRNA was synthesized as a negative control. Transfection was performed using Lipofectamine 3000 (Invitrogen, Carlsbad, CA, United States) according to the manufacturer’s instructions. Total RNA was collected 48 h after transfection. All the sequences used in the present study are the following: *BIRC5* siRNA: GAA​ACT​GCG​GAG​AAA​GTG​CGC. Scrambled siRNAs were used as negative control.

### 2.10 Cell Migration Assays

Cell migration assay was performed as previously described (Xiong et al., 2021). In this study, GBM cells, including U251 and A172 cells were utilized to determine the function of *BIRC5* on the LGG cell migration. Briefly, indicated cells were seeded into 6-well plates (2 × 10^6^/cell) and incubated for 1 day, and then a straight line was scraped with pipette tips. Detached cells were removed. Photographs were taken at the indicated time, and the relative traveled distance was measured. For the trans-well migration assay, 2 × 10^4^ cells/well in 100 μl serum-free medium were plated in a 24-well plate chamber insert, and the lower chamber was filled with 10 % FBS. After incubation for 24 h, cells were fixed with 4% PFA, washed, and then stained with 0.5% crystal violet for further pictures captured.

### 2.11 Immunohistochemistry Assay

Immunohistochemistry was performed as described previously (Xu et al., 2020). Briefly, paraffin sections were deparaffinized by xylene, rehydrated with gradient ethanol, and subjected to antigen retrieval. After H_2_O_2_ treatment and blockage with 10% normal goat serum, the slides were incubated with indicated antibodies (Proteintech, *BIRC5* Polyclonal Antibody, Rabbit Polyclonal,Catalog number: 10508-1-AP, 1:200) followed by an incubation with biotinylated secondary antibody and streptavidin-HRP (Dako, K5007).

### 2.12 Statistical Analysis

The survival data from the CGGA database were acquired by KM. Correlation analysis was performed using the Pearson correlation test. Kaplan-Meier survival curves were plotted to exhibit the overall survival for low-grade glioma patients. Univariate and multivariate Cox regression analyses were used to examine the independent prognostic significance of each variable enrolled in this finding. The significance of the data between the two experimental groups was determined by Student’s t-test, and multiple group comparisons were analyzed by one-way ANOVA. *p* < 0.05 (*), *p* < 0.01 (**) and *p* < 0.001 (***), were considered significant.

## 3 Results

### 3.1 Expression and Prognostic Value of *BIRC* Family Genes in Low-Grade Gliomas

To explore the functions of the *BIRC* gene family in LGG, we first examined the expression of *BIRC1–8* using the TCGA and GTEx datasets. *BIRC1-BIRC7* was found to be significantly up-regulated in LGG; BIRC8 genes showed stable expression levels, with no significant difference in expression between LGG and controls (Supplementary Figure S1A). We also used the GEPIA database to determine the prognostic value of *BIRC* family genes in LGG; among these genes, increased *BIRC5* expression was correlated with worse clinical outcomes in this cancer type (Supplementary Figure S1B). Overall, these results indicated that *BIRC5* was highly expressed in LGG with high specificity.

### 3.2 *BIRC5* is Highly Expressed in Human Cancer and is Related to Patient Prognosis

**FIGURE 1:**
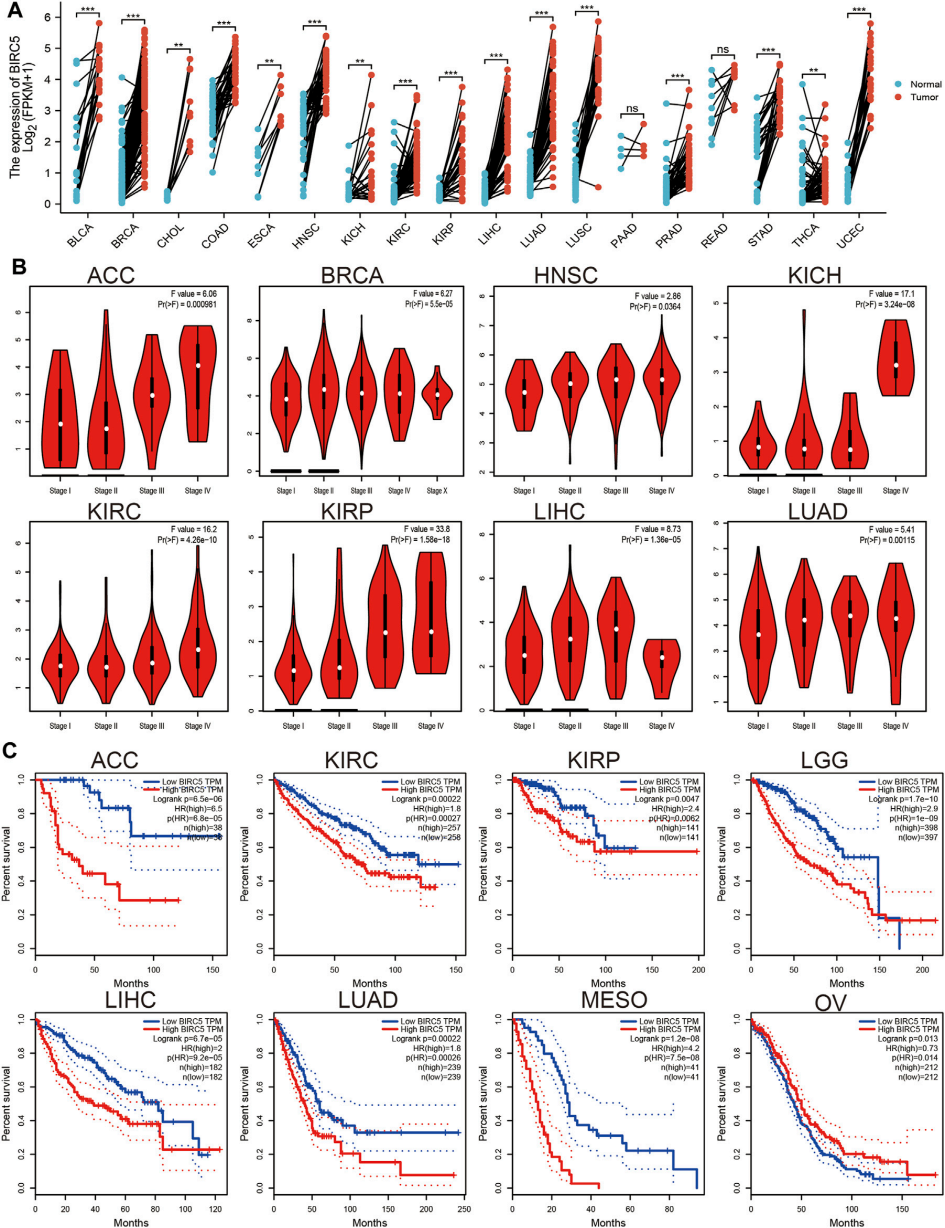
*BIRC5* expression and prognosis in patients with other cancer types. **(A)** Differences in expression of BIRC5in normal and tumor tissues of TCGA. **(B)** The pathological stage of BIRC5 in diverse cancer examine by UALCAN database. **(C)** Kaplan-Meier curves were drawn to evaluate the overall survival of *BIRC5* in various human cancers.

We used the TGCA database to examine the expression of *BIRC5* in all cancers. Our results demonstrated that *BIRC5* was significantly overexpressed in BLCA, BRCA, CHOL, COAD, ESCA, HNSC, KICH, KIRC, KIRP, LIHC, LUAD, LUSC, PRAD, READ, STAD, THCA, and UCEC (Figure 1A). Given that *BIRC5* was found to be highly expressed in diverse cancer types, we used the kmplot database to examine the prognostic value of *BIRC5* across human cancers. The analysis revealed that high levels of *BIRC5* expression closely correlated with worse clinical prognosis in ACC, KIRC, KIRP, LGG, LIHC, LUAD, and MESO. In contrast, high levels of *BIRC5* expression were associated with improved prognosis in OV (Figure 1B). Together, these findings indicate that *BIRC5* expression is closely associated with prognosis in diverse human cancers.

### 3.3 *BIRC5* is Highly Expressed in Glioma and is Related to Patient Prognosis

**FIGURE 2:**
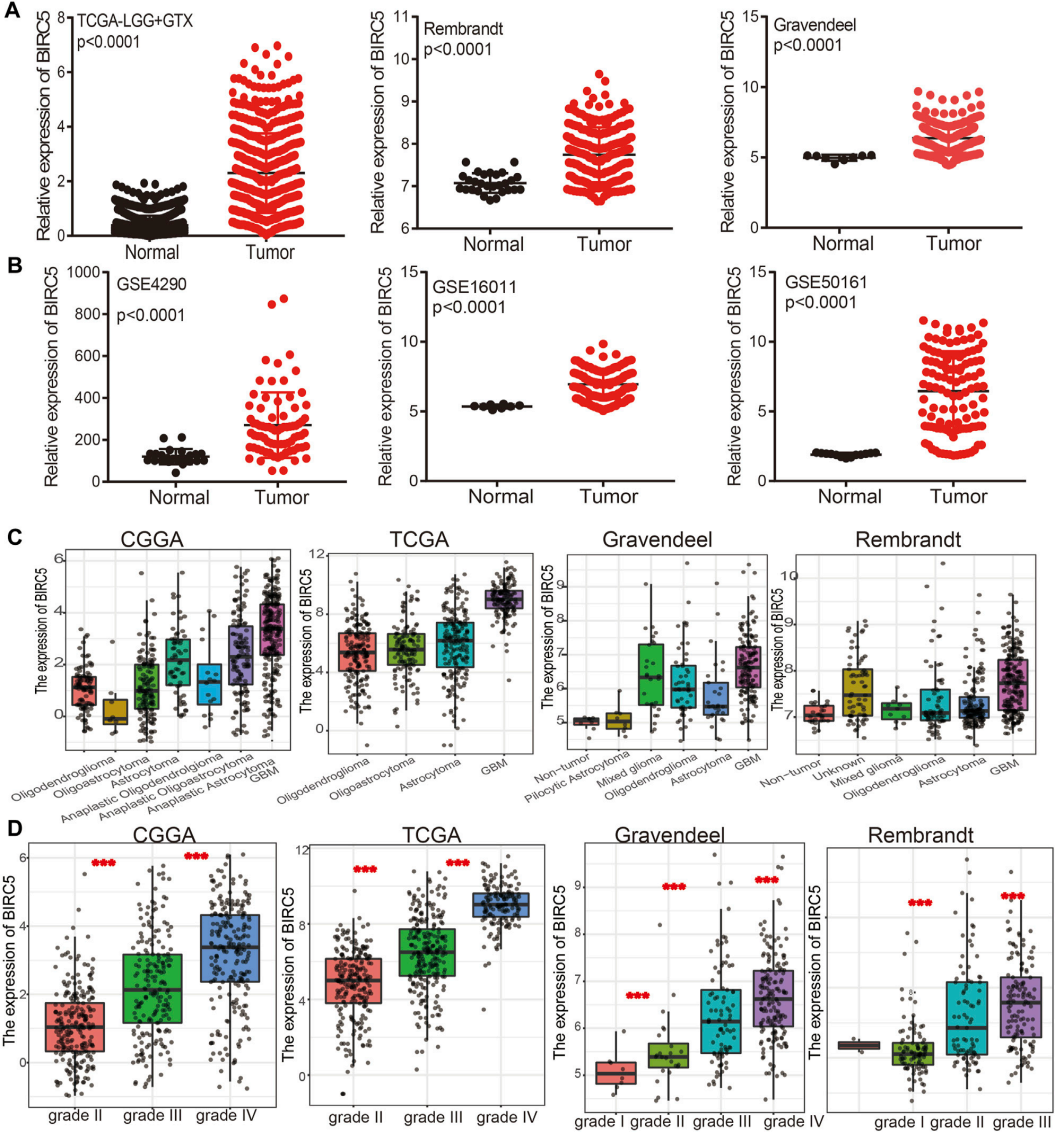
*BIRC5* was highly expressed in low grade glioma. **(A,B)** *BIRC5* was upregulated in glioma examined by the TCGA, Rembrandt, Gravendeel, and GEO datasets. **(C,D)** *BIRC5* expression levels among glioma patients with different histological subtype **(C)** and tumor grade **(D)** in TCGA-LGG.

To examine the expression of *BIRC5* in LGG tissue compared with a healthy control group, we used the public bioinformatics databases, including TCGA, GEO, Rembrandt, and Gravendeel. The results showed that *BIRC5* was significantly elevated in LGG tissue compared with controls (Figures 2A,B).

**FIGURE 3:**
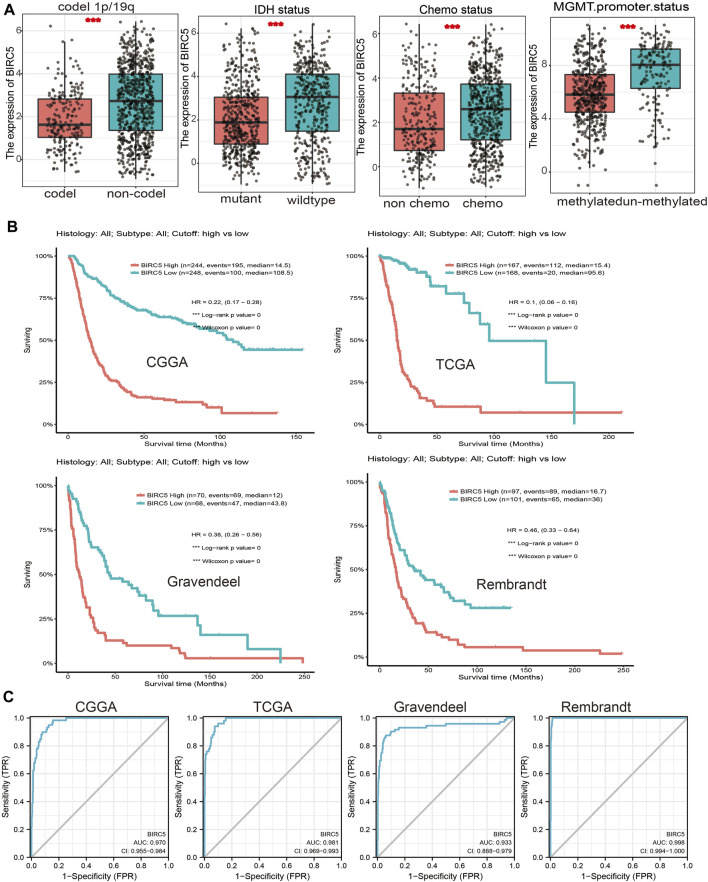
Correlation between the *BIRC5* expression and clinical information in LGG. **(A)** The expression of *BIRC5* in diverse clinical information of glioma based on CGGA databases. **(B)** The overall survival between the high- *BIRC5*and low- *BIRC5* in TCGA, CGGA, Rembrandt, and Gravendeel databases. **(C)** Diagnostic efficacy of the ROC curve of *BIRC5* expression in TCGA-LGG, CGGA, and TCGA-GBM.

We further analyzed the correlation between *BIRC5* expression levels and clinical characteristics of LGG, focusing primarily on tumor grade, histological subtype, chemotherapy status, IDH1 mutation, 1p/19q chromosomal co-deletion, and O[6]-methylguanine-DNA methyltransferase (MGMT) promoter methylation. Our results showed that *BIRC5* was differentially expressed in different histological subtypes of LGG (Figure 2C). *BIRC5* expression levels increased at higher tumor grades (Figure 2D). Interestingly, we found that *BIRC5* was markedly decreased in the group with IDH mutations and the group with 1p/19q chromosome co-deletion (Figure 3A and Table 1). Additionally, *BIRC5* expression was markedly increased in those receiving chemotherapy and those with unmethylated MGMT promoters. These results indicate that *BIRC5* plays a major role in LGG development.

**TABLE 1:** Correlation between *BIRC5* expression and clinicopathological factors based on the TCGA database.

Characteristic	Low expression of *BIRC5*	High expression of *BIRC5*	*p*
n	264	264	
WHO grade, *n* (%)			<0.001
G2	154 (33%)	70 (15%)	
G3	80 (17.1%)	163 (34.9%)	
IDH status, *n* (%)			<0.001
WT	27 (5.1%)	70 (13.3%)	
Mut	236 (45%)	192 (36.6%)	
1p/19q codeletion, *n* (%)			<0.001
codel	106 (20.1%)	65 (12.3%)	
non-codel	158 (29.9%)	199 (37.7%)	
Age, meidan (IQR)	38.5 (31, 49)	43 (33, 56)	0.005

As showed in the Table 2, patients having integral clinical data were included in further Cox regression analysis. In the Cox univariate regression analysis, we found that high expression of *BIRC5*, WHO grade, IDH status, 1p/19q codeletion, and age were associated with overall survival in LGG patients. Multivariate Cox analysis further indicated that *BIRC5* (*p* = 0.004) was an independent prognostic factor for overall survival in LGG patients, along with WHO grade, IDH status, 1p/19q codeletion, and age.

**TABLE 2:** Univariate and multivariate regression analyses of low-grade glioma.

Characteristics	Total(N)	Univariate analysis			Multivariate analysis	
		Hazard ratio (95% CI)	*p* Value		Hazard ratio (95% CI)	*p* Value
WHO grade	466					
G2	223					
G3	243	3.059 (2.046–4.573)	<0.001		1.756 (1.109–2.782)	0.016
IDH status	524					
WT	97					
Mut	427	0.186 (0.130–0.265)	<0.001		0.346 (0.218–0.549)	<0.001
1p/19q codeletion	527					
codel	170					
non-codel	357	2.493 (1.590–3.910)	<0.001		1.716 (1.020–2.889)	0.042
Histological type	332					
Oligoastrocytoma	134					
Oligodendroglioma	198	0.845 (0.527–1.352)	0.482			
Age	527					
<=40	264					
>40	263	2.889 (2.009–4.155)	<0.001		3.081 (2.015–4.711)	<0.001
*BIRC5*	527	1.464 (1.305–1.642)	<0.00		1.244 (1.072–1.443)	0.004

### 3.4 Prognostic Value of *BIRC5* in Low-Grade Gliomas

Given that *BIRC5* is highly expressed in LGG, we examined the prognostic value of *BIRC5* expression in this cancer type. People with LGG were divided into “high” or “low” expression groups based on the median expression value. High *BIRC5* expression was significantly associated with unfavorable prognosis in people with LGG in different datasets (Figure 3B). Receiver operator characteristic (ROC) curve analysis showed that the area under the curve (AUC) of *BIRC5* was 0.970 in the CGGA dataset, 0.981 in the TCGA-LGG dataset, 0.9933 in the Rembrandt dataset, and 0.998 in the Gravendeel dataset (Figure 3C). These results indicate that *BIRC5* is a highly sensitive and specific marker with the potential to be used in LGG diagnosis.

### 3.5 Correlation Between DNA Methylation and *BIRC5* Expression in Low-Grade Gliomas

**FIGURE 4:**
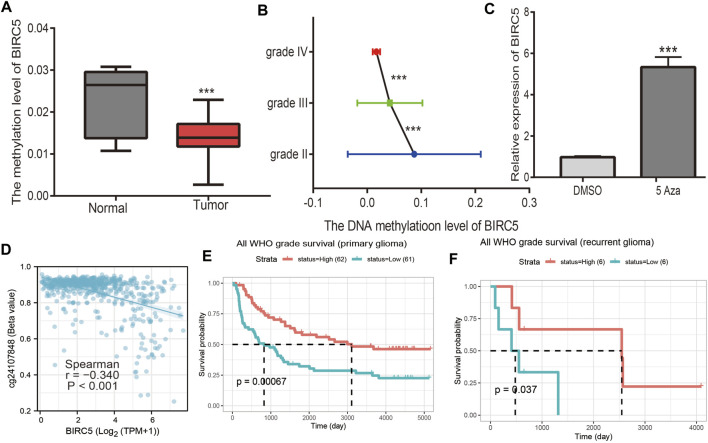
Analysis of the *BIRC5* methylation level. **(A)** The DNA methylation level of *BIRC5* in LGG. **(B)** The DNA methylation level of *BIRC5* in diverse tumor grades of LGG based on the CGGA dataset. **(C)** The expression of *BIRC5* in U251 cells after using 5-AZA treatment was examined by qRT-PCR assay. **(D)** The correlation between DNA methylation and *BIRC5* expression in low grade glioma by Pearson’s correlation. **(E,F)** The prognosis for methylation level of *BIRC5* in glioma based on the CGGA dataset.

DNA methylation is crucial for the epigenetic regulation of gene expression. To elucidate the mechanisms of abnormal upregulation of *BIRC5* in LGG tissues, we explored the correlation between DNA methylation and *BIRC5* expression in LGG using diverse public databases. Firstly, we found that *BIRC5* methylation was lower in LGG than in healthy brain tissue and was negatively correlated with tumor grade in LGG (Figures 4A,B). Treatment with 5-azacytidine, an inhibitor of DNA methyltransferase (Xiong et al., 2021), resulted in increased *BIRC5* RNA levels in U251 human brain cells (Figure 4C). Moreover, we found that cg24107848 methylation was significantly negatively correlated with the *BIRC5* expression in LGG (r = −0.340, *p* < 0.001) (Figure 4D). We also showed that decreased methylation levels at cg28734000 correlated with worse prognosis in the CGGA-LGG dataset using the MethSurv statistical tool (Figures 4E,F). These results suggested that hypomethylation in the *BIRC5* promoter region results in increased expression of this gene in LGG.

### 3.6 *BIRC5* Expression is Correlates with Regulators of N6-methyladenosine (m6A) RNA Methylation in Low-Grade Gliomas

**FIGURE 5:**
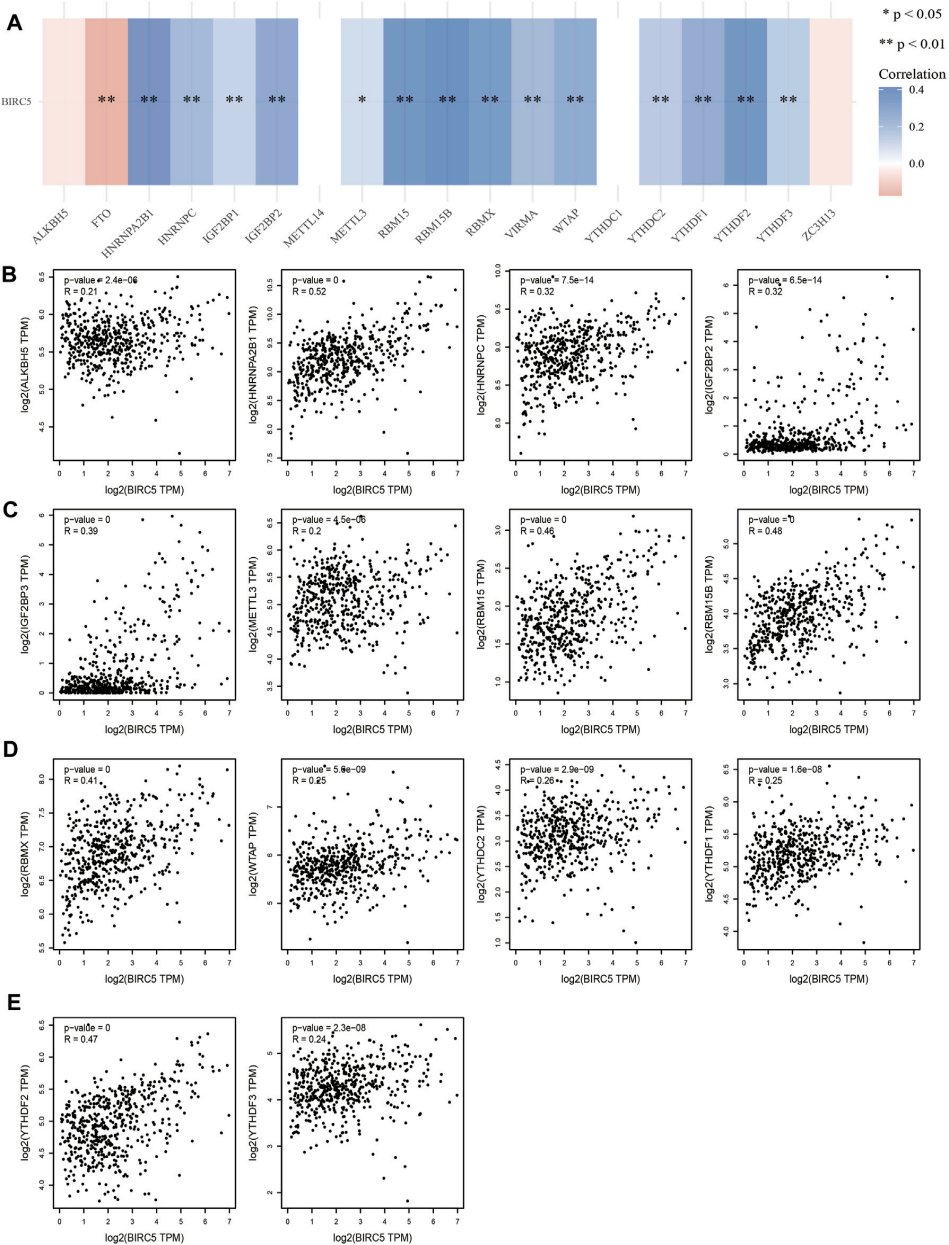
Analysis of the correlation between *BIRC5* expression and m6A regulator in LGG. **(A–E)** Analysis of the correlation between *BIRC5* expression and m6A regulator in low grade glioma by Pearson’s correlation.

Given that N6-methyladenosine (m6A) RNA methylation plays a major role in the initiation and progression of LGG, we examined the correlation between *BIRC5* expression and regulators of m6A RNA methylation in this cancer type (Figure 5A). *BIRC5* expression was significantly positively correlated with 16 m6A-related genes, including HNRNPA2B1 (r = 0.52, *p* = 0), HNRNPC (r = 0.0.32, *p* < 0.001), IGF2BP2 (r = 0.32, *p* <0.001), IGF2BP3 (r = 0.39, *p* = 0), METTL3 (r = 0.20, *p* < 0.001), RBM15 (r = 0.46, *p* = 0), RBM15B (r = 0.48, *p* = 0), RBMX (r = 0.41, *p* = 0), WTAP (r = 0.25, *p* < 0.001), YTHDC2 (r = 0.26, *p* < 0.001), ALKBH5 (r = 0.21, *p* = 0.001), YTHDF1 (r = 0.25, *p* = 0.001), YTHDF2 (r = 0.47, *p* = 0) and YTHDF3 (r = 0.24, *p* = 0.001) (Figures 5B–E). Collectively, these results demonstrate that *BIRC5* plays indispensable roles in LGG progression via interaction with regulators of m6A RNA methylation.

### 3.7 Functional Analysis of *BIRC5* in Low-Grade Gliomas

**FIGURE 6:**
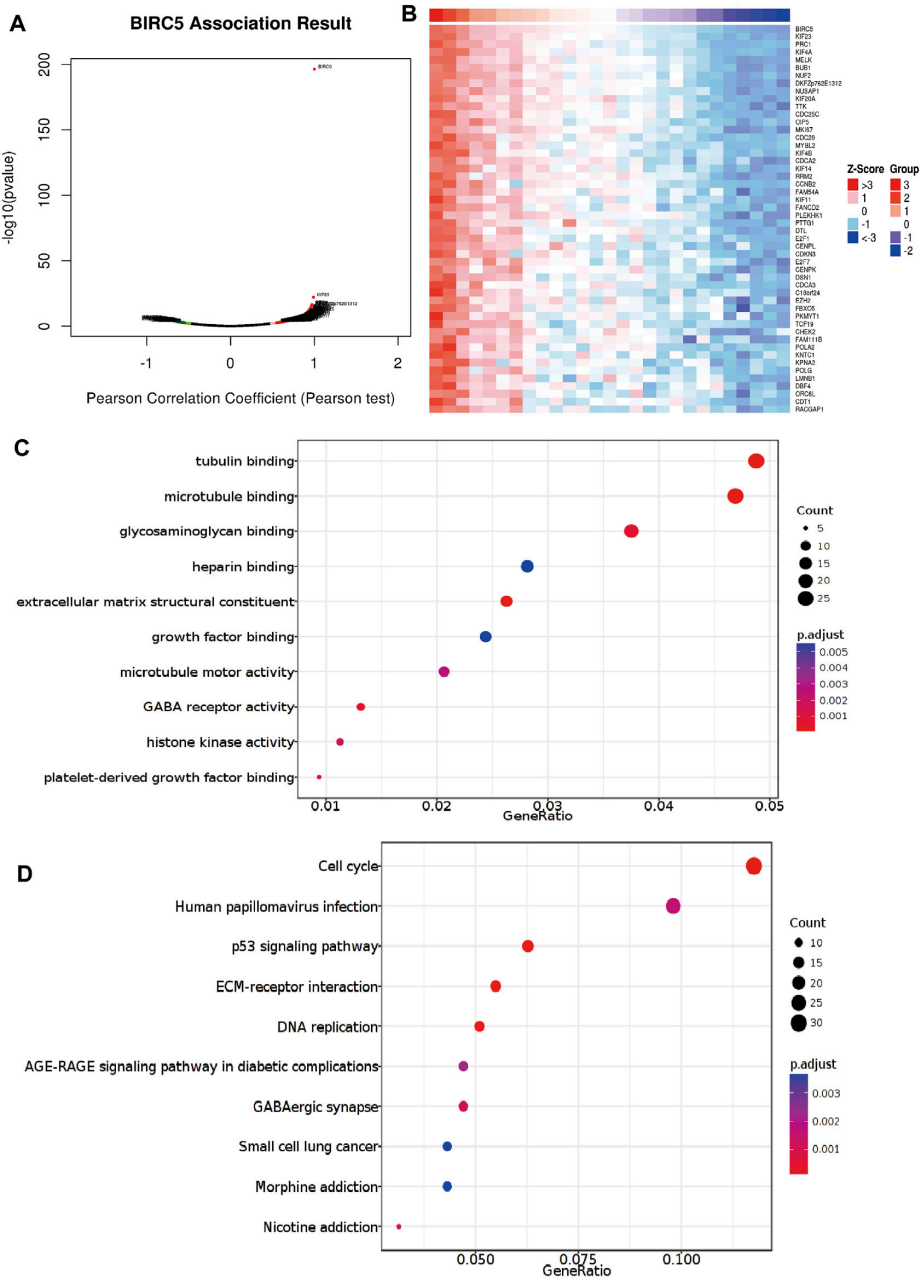
Analysis of the function of *BIRC5* expression in LGG. **(A,B)** Analysis of the co-expression gene of *BIRC5* in LGG examines by linkomics database. **(C)** Analysis of the molecular function of *BIRC5* in LGG. **(D)** Analysis of the KEGG signaling pathway of *BIRC5* in LGG.

To explore the specific functions of *BIRC5* in LGG progression, we first analyzed genes whose expression was positively correlated with that of *BIRC5* in LGG (Figures 6A,B). We then performed Kyoto Encyclopedia of Genes and Genomes (KEGG) and Gene Ontology (GO) enrichment analyses based on these co-expressed genes. The results showed that *BIRC5* is primarily involved in tubulin, microtubule, glycosaminoglycan, heparin, and growth factor binding, as well as GABA receptor activity and histone activity in the GO analysis (Figure 6C). *BIRC5* may participate in cell cycle, human papillomavirus infection, p53 signaling, extracellular matrix (ECM) receptor interaction, DNA replication, small cell lung cancer, and morphine addiction pathways (Figure 6D). These results demonstrate functionally-relevant roles for *BIRC5* in LGG progression.

### 3.8 *BIRC5*-Related Signaling Pathways Identified Using Gene Set Enrichment Analysis

**FIGURE 7:**
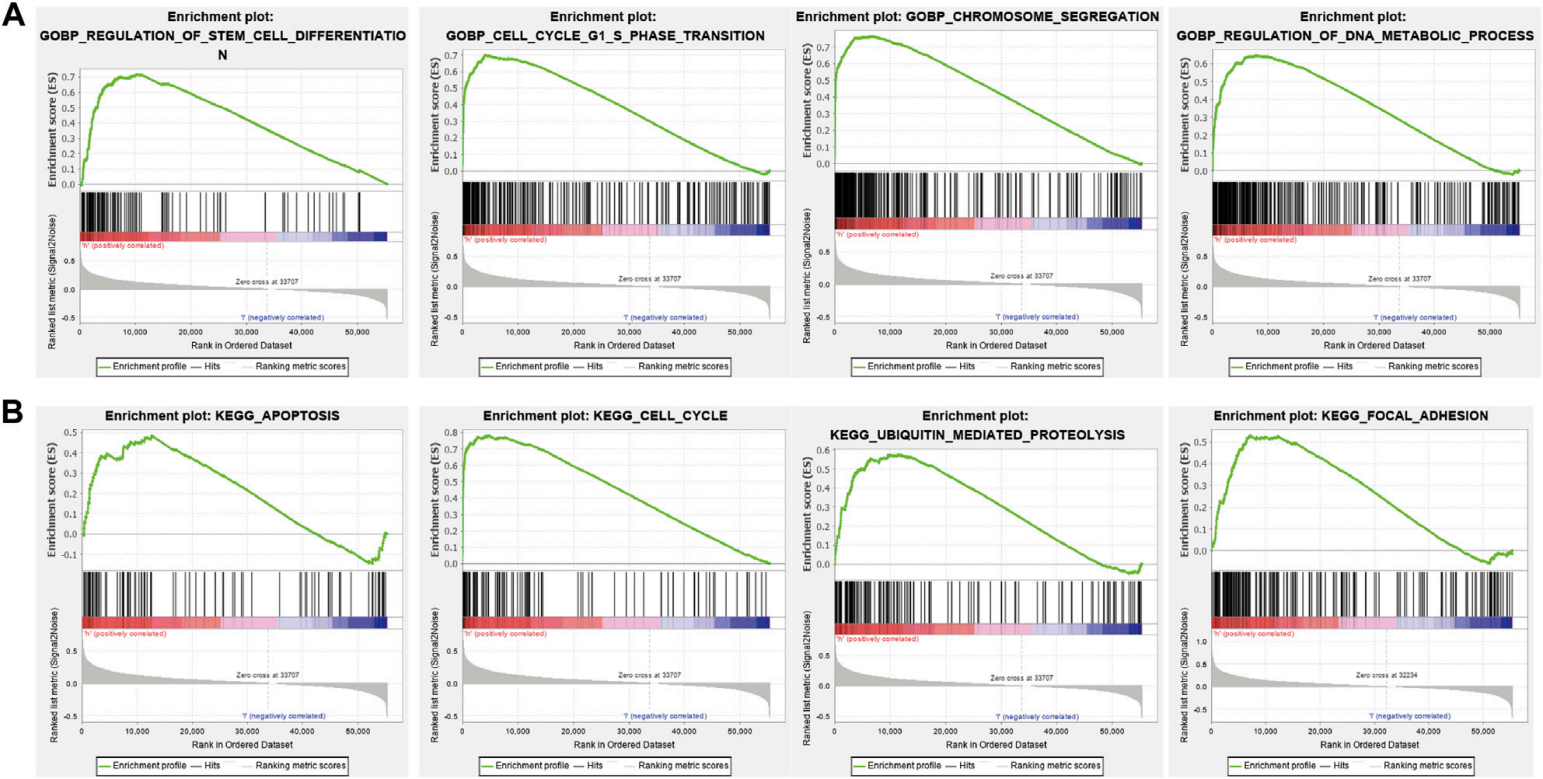
The KEGG signal pathway explored by GSEA software. **(A)** The biological process involved by *BIRC5* in LGG was examined by GSEA software. **(B)** The signaling pathway involved by *BIRC5* in LGG was examined by GSEA software.

To identify signaling pathways that are affected by *BIRC5* overexpression in glioma, we performed a gene set enrichment analysis (GSEA). High *BIRC5* expression was mainly associated with the regulation of stem cell differentiation, cell cycle growth 1/synthesis (G1/S) phase transition, and chromosome segregation (Figure 7A). Moreover, high *BIRC5* expression was associated with apoptosis, cell cycle, ubiquitin-mediated proteolysis, and focal adhesion signaling pathways (Figure 7B). Collectively, these results demonstrate that *BIRC5* plays an indispensable role in cell proliferation and immune regulation.

### 3.9 Association Between *BIRC5* Expression Levels and Immune Cell Infiltration in Low-Grade Gliomas

**FIGURE 8:**
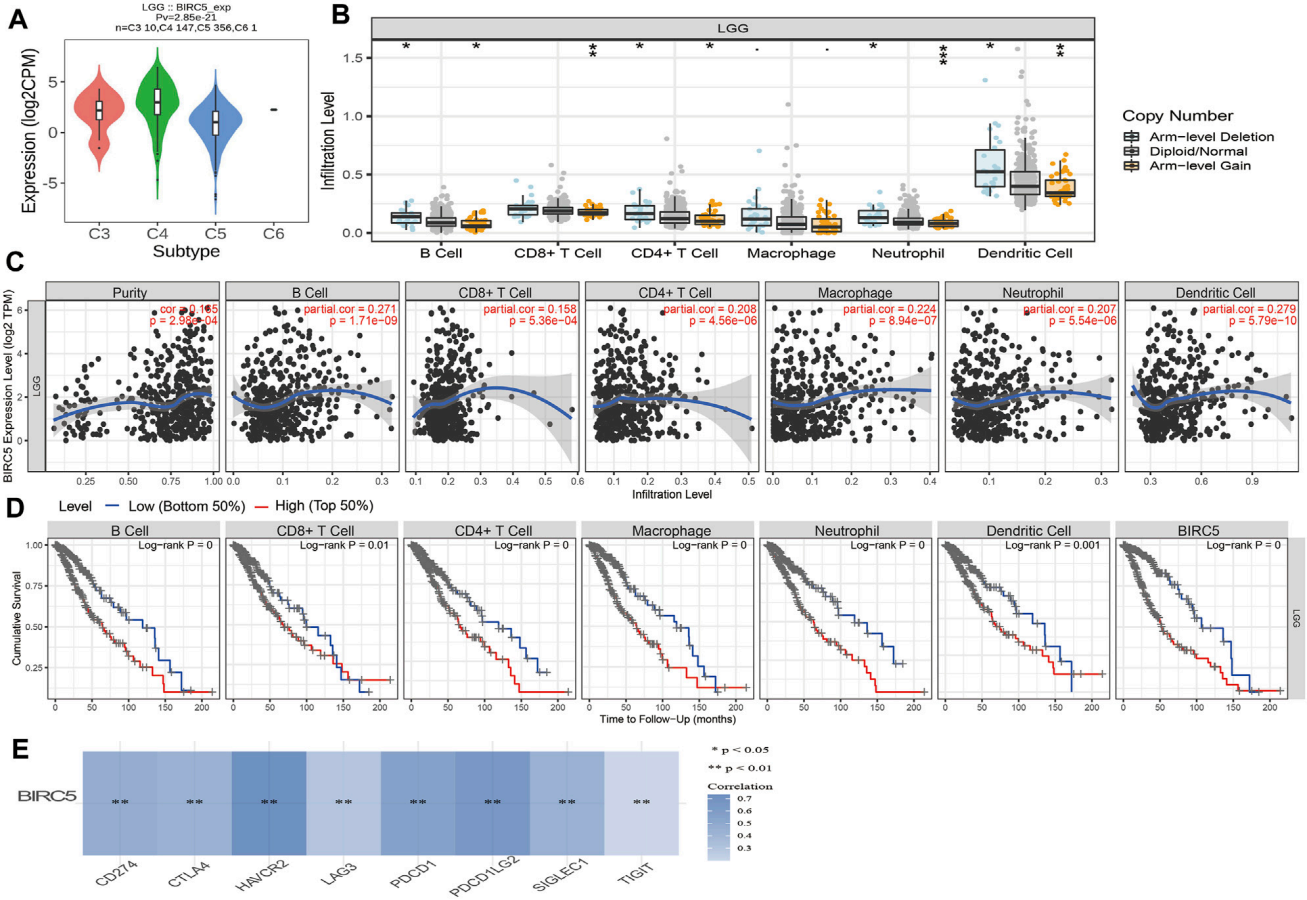
Analysis of the correlation between *BIRC5* expression and diverse immune cell infiltration. **(A)** The expression of *BIRC5* in diverse immune subtypes of LGG was examined by TISIDB. **(B)** The correlation between *BIRC5* expression and somatic copy number alterations examine by TIMER database. **(C)** The correlation between *BIRC5* expression and diverse immune cell infiltration was examined by TIMER database. **(D)** The B cells, CD4^+^ T cells, CD8^+^ T cells, dendritic cells, Macrophages, and Neutrophils are correlated with the cumulative survival rate in LGG examine by TIMER database. **(E)** The correlations between *BIRC5* expression and diverse immune checkpoints related gene examined by TIMER database.

The level of immune infiltration affects clinical outcomes from tumors. We, therefore, analyzed the expression of *BIRC5* in diverse immune subtypes, which showed that *BRIC5* expression was highest in the C4 subtype (Figure 8A). Furthermore, we found that different somatic *BIRC5* copy number alterations affected infiltration levels of diverse immune cells, including B cells, CD4^+^ and CD8^+^ T cells, neutrophils, and dendritic cells (Figure 8B). Finally, we using the TIMER database to examine the correlation between *BIRC5* expression and immune infiltration levels for diverse immune cells in LGG; results indicated that *BIRC5* was significantly correlated with the level of B cells (cor = 0.271 *p* < 0.001), CD8^+^ T cells (cor = 0.158, *p* < 0.001), CD4^+^ T cells (cor = 0.208, *p* < 0.001), macrophages (cor = 0.224, *p* < 0.001), neutrophils (cor = 0.207, *p* < 0.001), and dendritic cells (cor = 0.279, *p* < 0.001), and positively correlated with tumor purity (cor = 0.165, *p* < 0.001) (Figure 8C). Our Cox proportional hazard model analysis demonstrated that B cells, CD8^+^ and CD4^+^ T cells, macrophages, neutrophils, dendritic cells, and *BIRC5* expression were significantly associated with worse overall survival in LGG patients (Figure 8D).

Given that immune checkpoints play a crucial role in tumor immunosuppression, we analyzed the correlation between *BIRC5* expression and that of the immune checkpoint-related genes *CD274, CTLA4, HAVCR2, LAG3, PDCD1, PDCD1LG2, TIGIT*, and *SIGLEC15* in LGG using Pearson correlation analysis. *BIRC5* expression was significantly positively correlated with the expression of all eight genes in this analysis (Figure 8E), supporting a role for *BIRC5* in immune regulation.

### 3.10 Correlation Between *BIRC5* Expression and Drug Sensitivity

**FIGURE 9:**
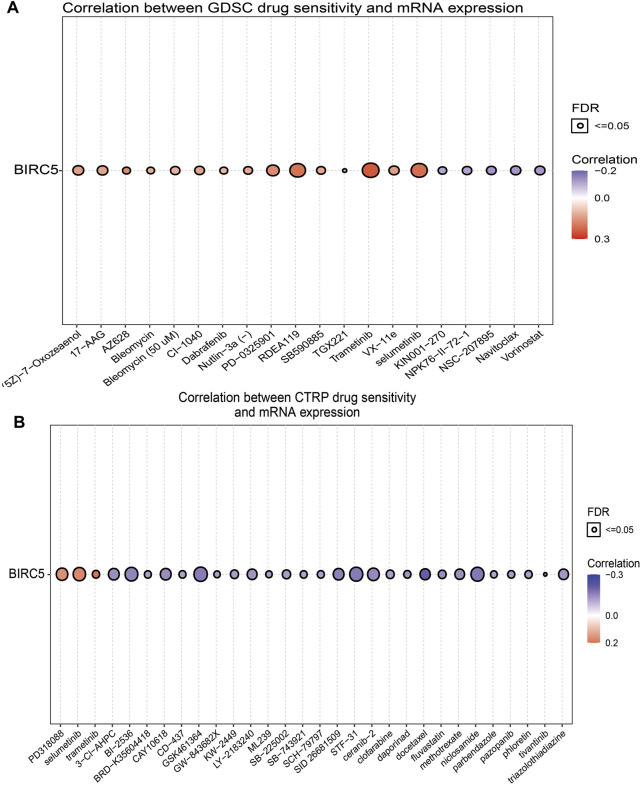
Correlation between drug sensitivity and *BIRC5* expression. **(A,B)** The correlation between *BIRC5* expression and drug sensitivity in pan-RCC was studied by Spearman correlation analysis. Red represents positive correlation between drugs sensitivity and *BIRC5* expression, and blue represents negative correlation between drugs sensitivity and *BIRC5* expression. **(A)**: GDSC, **(B)**: CTRP.

Given that *BIRC5* has potentially promoted LGG progression, it is therapeutically useful to identify anti-LGG drugs targeting *BIRC5*. Therefore, we used GSCA tools to analyze the relationship between *BIRC5* expression and sensitivity to anti-LGG drugs. *BIRC5* expression was positively correlated with drug sensitivity for trametinib, selumetinib, RDEA119, AZ628, PD-0325901, and VX-11e (all r > 0.14, *p* < 0.001), and negatively associated with drug sensitivity for NSC-207895, navitoclax, vorinostat, NPK76-II-72-1, and KIN001-270 (all r < −0.10, *p* < 0.001) in the Genomics of Drug Sensitivity to Cancer (GDSC) database (Figure 9A and Table 3). Similarly, CTRP database analysis demonstrated that *BIRC5* expression was positively correlated with drug sensitivity for trametinib, selumetinib, PD318088, MLN2480, BYL-719, PD 153035 and GANT-61 (all r > 0.10, *p* < 0.001), and negatively associated with the drug sensitivity of BRD-K30748066, docetaxel, GSK-J4, niclosamide, STF-31, GSK461364, SID 26681509, tivantinib, BI-2536, ceramic-2, neopeltolide, CAY10618, fluvastatin, 3-Cl-AHPC and methotrexate (r < −0.14, *p* < 0.001) (Figure 9B and Table 4). Taken together, the results demonstrate that *BIRC5* was correlated with sensitivity to diverse drugs from the Cancer Therapeutic Response Portal database.

**TABLE 3:** Analysis of the correlation between *BIRC5* expression and diverse drug sensitivity by using GDSC database.

Symbol	Drug	cor	fdr
*BIRC5*	Trametinib	0.243081	2.28E-12
*BIRC5*	selumetinib	0.211106	7.21E-10
*BIRC5*	RDEA119	0.204072	2.52E-09
*BIRC5*	AZ628	0.169564	0.007743
*BIRC5*	PD-0325901	0.168231	6.72E-06
*BIRC5*	VX-11e	0.141613	0.000448
*BIRC5*	NSC-207895	−0.12988	0.001041
*BIRC5*	Navitoclax	−0.12844	0.000445
*BIRC5*	Vorinostat	−0.12516	0.000561
*BIRC5*	NPK76-II-72-1	−0.11152	0.001414
*BIRC5*	KIN001-270	−0.1075	0.00422

**TABLE 4:** Analysis of the correlation between *BIRC5* expression and diverse drug sensitivity by CTRP database.

Symbol	Drug	cor	fdr
*BIRC5*	trametinib	0.198327	0.002034
*BIRC5*	selumetinib	0.172965	4.03E-05
*BIRC5*	PD318088	0.1609	0.000102
*BIRC5*	MLN2480	0.141203	0.086218
*BIRC5*	BYL-719	0.122098	0.037759
*BIRC5*	PD 153035	0.116199	0.029237
*BIRC5*	GANT-61	0.102086	0.051344
*BIRC5*	BRD-K30748066	−0.30066	0.067445
*BIRC5*	docetaxel	−0.20703	0.000309
*BIRC5*	GSK-J4	−0.185	0.245138
*BIRC5*	niclosamide	−0.17165	2.12E-05
*BIRC5*	STF-31	−0.16615	2.02E-05
*BIRC5*	GSK461364	−0.16341	1.45E-05
*BIRC5*	SID 26681509	−0.15853	0.000199
*BIRC5*	tivantinib	−0.15799	0.004965
*BIRC5*	BI-2536	−0.15406	2.91E-05
*BIRC5*	ceranib-2	−0.14683	8.62E-05
*BIRC5*	neopeltolide	−0.14328	0.116341
*BIRC5*	CAY10618	−0.14301	0.000196
*BIRC5*	fluvastatin	−0.14245	0.00154
*BIRC5*	3-Cl-AHPC	-0.14163	0.000201
*BIRC5*	methotrexate	−0.14093	0.000493

### 3.11 Knockdown of *BIRC5* Inhibits Glioma Cell Migration *In Vitro*

**FIGURE 10:**
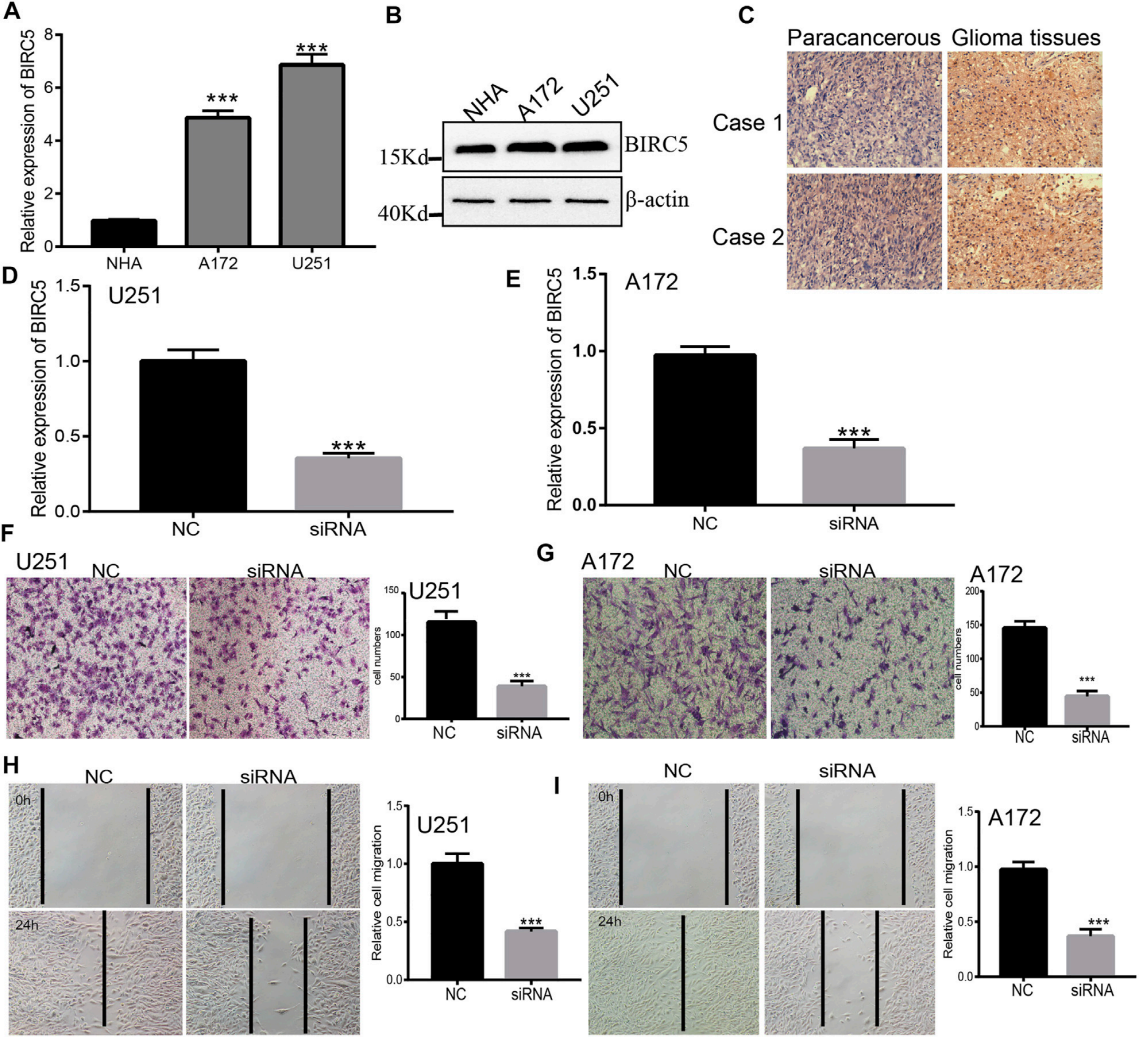
Depletion of *BIRC5* inhibits glioma cells migration. **(A,B)** The expression of *BIRC5* in normal human astrocytes cells (NHA) and glioma cell lines (A172 and U251) examined by qRT-PCR and Western blot assay. **(C)** IHC analysis of *BIRC5* expression in glioma cancer tissues. Scale bar, 50 μm. **(D,E)** The expression of *BIRC5* in U251 and A172 cells treated with siRNAs (siRNA -*BIRC5*#NC, siRNA-*BIRC5*#) measured by qRT–PCR. *p < 0.05, one-way ANOVA test for the comparison of the two groups. **(F–I)** Transwell and Wound healing assay of the glioma cancer cell lines U251 and A172 treated with shRNAs. *p < 0.05, one-way ANOVA test for the comparison of the two groups. Scale bar, 100 μm.

Our KEGG enrichment results showed that *BIRC5* may be involved in the focal adhesion signaling pathway, therefore, we used a functional assay to verify this result. We used a quantitative real-time polymerase chain reaction (qRT-PCR) and Western blot assay to detect *BIRC5* expression in normal human astrocyte cells and glioma cell lines. *BIRC5* was highly expressed in glioma cell lines, especially in U251 cells (Figures 10A,B). IHC assay results also indicated that *BIRC5* was highly expressed in glioma tissues than paracancerous tissues (Figure 10C). Based on this result, we constructed *BIRC5* knockdown cells using qRT-PCR to verify knockdown efficiency (Figures 10D,E). The resultant depletion of *BIRC5* was found to significantly inhibit glioma cell migration by transwell and wound healing assays (Figures 10F–I). Collectively, these results demonstrate that *BIRC5* is highly expressed in glioma cells, likely resulting in increased cell migration.

## 4 Discussion

Glioma is a highly fatal primary tumor of the central nervous system. Despite diverse available therapeutic strategies, including surgery, chemotherapy, and immunotherapy, the median survival of people with glioma—which has barely improved since its recognition as a separate cancer type—remains only approximately 14  months (Huang et al., 2021). Therefore, more sensitive and specific diagnostic biomarkers and potential therapeutic targets for glioma patients are of no delay.

*BIRC5*, also called survivin, is an immune-related gene and member of the apoptotic (IAP) protein family (Li et al., 1998). *BIRC5* has been shown to play vital roles in carcinogenesis by affect the cell proliferation and by inhibits cell apoptosis (Yamamoto et al., 2008). Since *BIRC5* is frequently up-regulated in diverse cancers and treatment that targets *BIRC5* has been increasingly noticed as a novel strategy for various malignant tumors (Jaiswal et al., 2015). For instance, *BIRC5* was increased in triple-negative and *BIRC5* knockdown significantly inhibits the proliferation of breast cancer cells, implying that *BIRC5* acts like a tumor driver (Wang et al., 2012). Furthermore, *BIRC5* expression has been found to confer resistance to chemotherapy and radiation, targeting *BIRC5* in experimental models improves survival (Jha et al., 2012). Together, these findings indicate that *BIRC5* may not only function as an oncogene, but also as a promising predictive biomarker and potential therapeutic target in cancer. A recent study confirmed that *BIRC5* could act as a reliable prognostic indicator in breast cancer patients (Dai et al., 2020), but the expression, clinical significance, and its impact on the immune microenvironment remain largely unclear in low-grade glioma.

Brunicardi et al. found that *BIRC5* was highly expressed in pancreatic cancer and maybe as a precision diagnostic biomarker for the diagnosis for pancreatic cancer. In this study, we uncover that *BIRC5* expression was upregulated in diverse human cancer and correlated with poor prognosis, especially in glioma (Figure 1). Which further validated by using the GEO and CGGA database (Figure 2), the IHC and Western blotting assay in glioma cell lines and glioma tissues. Furthermore, higher expression of *BIRC5* was positively correlates with adverse clinicopathologic features of glioma, including the tumor grade, histological subtype, isocitrate dehydrogenase 1 (IDH1) mutation, 1p/19q chromosomal co-deletion, chemotherapy status, and O[6]-methylguanine-DNA methyltransferase (MGMT) promoter methylation status (Figure 3). Accumulating studies have confirmed that IDH1 mutations are associated with the occurrence and development of glioma, and IDH1 mutations are more common in low grade glioma than in LGG (Miroshnikova et al., 2016). Therefore, we speculated that the higher expression of *BIRC5* in low grade glioma patients might due to more low grade glioma patients are IDH wild-type tumors, which expressed higher *BIRC5* levels. Moreover, the KM survival curve analyses showed that upregulated *BIRC5* expression associated with adverse clinical outcomes in glioma from both TCGA and CGGA datasets. ROC curve analysis showed that the *BIRC5* expression level has diagnostic value for gliomas (Figure 3). Similarly, high expression of *BIRC5* was associated with overall survival in breast cancer, clear-cell renal cell carcinoma, gastric cancer, and lung cancer (Ni et al., 2019; Wan et al., 2019; Zou et al., 2019; Dai et al., 2020). We also performed a univariate analysis including the prognostic factors for overall survival. We found that high expression of *BIRC5* was associated with higher tumor grades, histological type, *IDH* mutation status, 1p/19q chromosome co-deletion, and primary therapy outcome. Furthermore, we utilized the Cox regression model for multivariate analysis; the results demonstrated that *BIRC5* expression, WHO grade, IDH status, 1p/19q codeletion, and age were an independent prognostic factor for overall survival in LGG patients. Similarly, high expression of *BIRC5* was an independent prognostic factor for overall survival in clear-cell renal cell carcinoma and endometrial cancer (Chuwa et al., 2016; Wan et al., 2019).

A growing number of studies have reported that DNA methylation plays an important role in gene expression regulation (Yamashita et al., 2018). In this study, we found that the DNA methylation level of *BIRC5* was down-regulated in glioma tissues than normal tissues. Furthermore, we showed that the methylation sites (cg24107848) were significantly negatively correlated with the expression of *BIRC5* in LGG. More importantly, our results showed that lower methylation levels in cg24107848 were correlated with a worse prognosis in LGG patients. We also used a qRT-PCR assay confirm that hypomethylation in the *BIRC5* promoter region resulted in the increased expression of this gene in LGG (Figure 4). Although various molecular mechanisms can lead to increased gene expression, such as the lncRNA/miRNA axis, regulation of transcription factors, and gene copy number amplification, DNA hypomethylation is one of the main regulatory mechanisms of gene expression.

M6A RNA methylation is also known to affect glioma malignancy (Chai et al., 2019). For example, it has been shown that YTHDF2 promotes the CSC liver phenotype and cancer metastasis by modulating the m6A methylation of OCT4 mRNA (Zhang et al., 2020). In hepatocellular carcinoma, YTHDF2 silenced in human HCC cells or ablated in mouse hepatocytes provoked inflammation, vascular reconstruction, and metastatic progression (Hou et al., 2019). A recent finding found that YTHDF2 sequesters m6A-circRNA and is essential for suppression of innate immunity (Chen et al., 2019). In this study, we found that *BIRC5* expression is significantly associated with regulators of m6A methylation (Figure 5). Crosstalk between m6A RNA modification and *BIRC5* expression in low-grade glioma, therefore, requires further characterization in future studies.

Sun et al. found that *BIRC5* was highly expressed in HCC tissues and involved in cell cycle regulation (Wang et al., 2011). To explore the possible mechanism of *BIRC5* affecting the survival of patients, KEGG and GSEA enrichment analyses were performed based on the differentially expressed genes between *BIRC5* groups, and the results suggested that higher *BIRC5* expression was mainly associated with the regulation of stem cell differentiation, cell cycle growth 1/synthesis (G1/S) phase transition, and chromosome segregation (Figure 6), which is consistent with previous studies that *BIRC5* regulated the stem cell differentiation (Lin et al., 2020). Moreover, high *BIRC5* expression was associated with apoptosis, cell cycle, ubiquitin-mediated proteolysis, and focal adhesion signaling pathways. Collectively, these results demonstrate that *BIRC5* plays an indispensable role in cell proliferation and immune regulation (Figure 7).

Mounting evidence demonstrates that the tumor microenvironment has a key role in the development of tumors, and can be used as a biomarker for diagnosis and prognosis (Wu and Dai, 2017). CD8 + tumor infiltrating lymphocytes have also been shown to be associated with glioma prognosis (Yu-Ju Wu et al., 2020). In this research, we revealed that *BIRC5* somatic copy number alterations affected infiltration levels of various immune cells, including B cells, CD4^+^ and CD8^+^ T cells, neutrophils, and dendritic cells. Furthermore, we found that *BIRC5* was significantly correlated with the level of B cells, CD8^+^ T cells, CD4^+^ T cells, macrophages, neutrophils and dendritic cells, and was negatively correlated with tumor purity. We showed that *BIRC5* influences the survival time of LGG patients, partially through immune cell infiltration (Figure 8). These findings indicate that *BIRC5* could be a novel immune-related therapeutic target in LGG.

It has been demonstrated that immune modulators play crucial roles in tumor cell evasion of immune surveillance (Xue et al., 2017). Studies have confirmed that the increased of immune checkpoints such as PD-L1, CTLA-4, TIM-3, and LAG3 in glioma helps tumor immune evasion, leading to T cell dysfunction (Xue et al., 2017; Woroniecka et al., 2018). Our results confirmed that *BIRC5* expression was significantly positively correlated with the expression of CD274, CTLA4, HAVCR2, LAG3, PDCD1, PDCD1LG2, TIGIT, and SIGLEC15, suggesting a crucial role of *BIRC5* in regulating the expression of immune checkpoints and immunotherapy (Figure 8). Given the effects of *BIRC5* on LGG immune cell infiltration, we can infer that increased expression of *BIRC5* may promote mast cell infiltration and contribute to a poor prognosis. Therefore, our results demonstrated that *BIRC5* might affect immune cell infiltration, making them a predictive biomarker for immunotherapy in LGG patients. Finally, we found that *BIRC5* was correlated with sensitivity to diverse drugs in the cancer therapeutic response portal database, with some positive and some negative correlations (Figure 9). For the biological function on the glioma cells, we found that *BIRC5* was highly expressed in glioma cell lines, depletion of *BIRC5* significantly inhibiting the migration of glioma cells (Figure 10).

In summary, we found that *BIRC5* was significantly aberrantly overexpressed in glioma tissues and cells. Through a series of comprehensive approaches, we demonstrated that the upregulated *BIRC5* expression is strongly associated with clinicopathologic features, adverse clinical outcomes, and immune cell infiltration in low-grdae glioma. Altogether, our results suggest that *BIRC5* might serve as a valuable prognostic factor and a promising novel immunotherapy target for glioma.

This study improves our understanding of the correlation between *BIRC5* and low-grade glioma, but some limitations still exist. First, although we explored the correlation between *BIRC5* and immune infiltration in LGG patients, there is a lack of experiments to validation the function of *BIRC5* in the tumor microenvironment regulation of LGG. Second, we uncover that depletion of *BIRC5* was inhibits cell migration of LGG cells. However, the potential molecular mechanisms of *BIRC5* in tumor metastasis need to be explored in further studies. Third, we did not conduct the *in vivo* experiments to validation the function of *BIRC5* in the tumor metastasis and tumor microenvironment regulation of LGG. In the future, we will pay more attention to the function of *BIRC5* in tumor metastasis and tumor microenvironment regulation of LGG. Furthermore, we will perform more *in vivo* and vitro experiments to explore the function and the potential molecular mechanisms of *BIRC5* in tumor metastasis and tumor microenvironment regulation of LGG.

Overall, our results confirmed that *BIRC5* could serve as a potential novel prognostic biomarker for LGG. Moreover, we explored the underlying evidence indicating that *BIRC5* regulates immune cell infiltration in the tumor microenvironment in LGG patients. Therefore, these findings are potentially valuable in advancing our current understanding of not only the role of *BIRC5* but also its translational use in LGG prognosis and immunotherapy.

## Conclusion

Our study found that *BIRC5* expression was increased in low-grade glioma, which was also associated with poor prognosis. Furthermore, low-grade glioma might be involved in the initiation and progression of LGG by regulating the function of immune infiltrating cells and cell migration. Herein, we revealed the biological functions of *BIRC5* in LGG and offered a potential strategy for the diagnosis and treatment of LGG.

## Data Availability

The original contributions presented in the study are included in the article/Supplementary Material, further inquiries can be directed to the corresponding authors.
