# Supplementary material for: The N6-Methylandenosine-Related Gene BIRC5 as a Prognostic Biomarker Correlated With Cell Migration and Immune Cell Infiltrates in Low Grade Glioma
Source: Front Mol Biosci. 2022 Mar 3;9:773662. doi: 10.3389/fmolb.2022.773662 (PMC8927544; doi:10.3389/fmolb.2022.773662)
Supplement: Supplementary file 1 [file DataSheet1.docx]

Supplementary Material

**
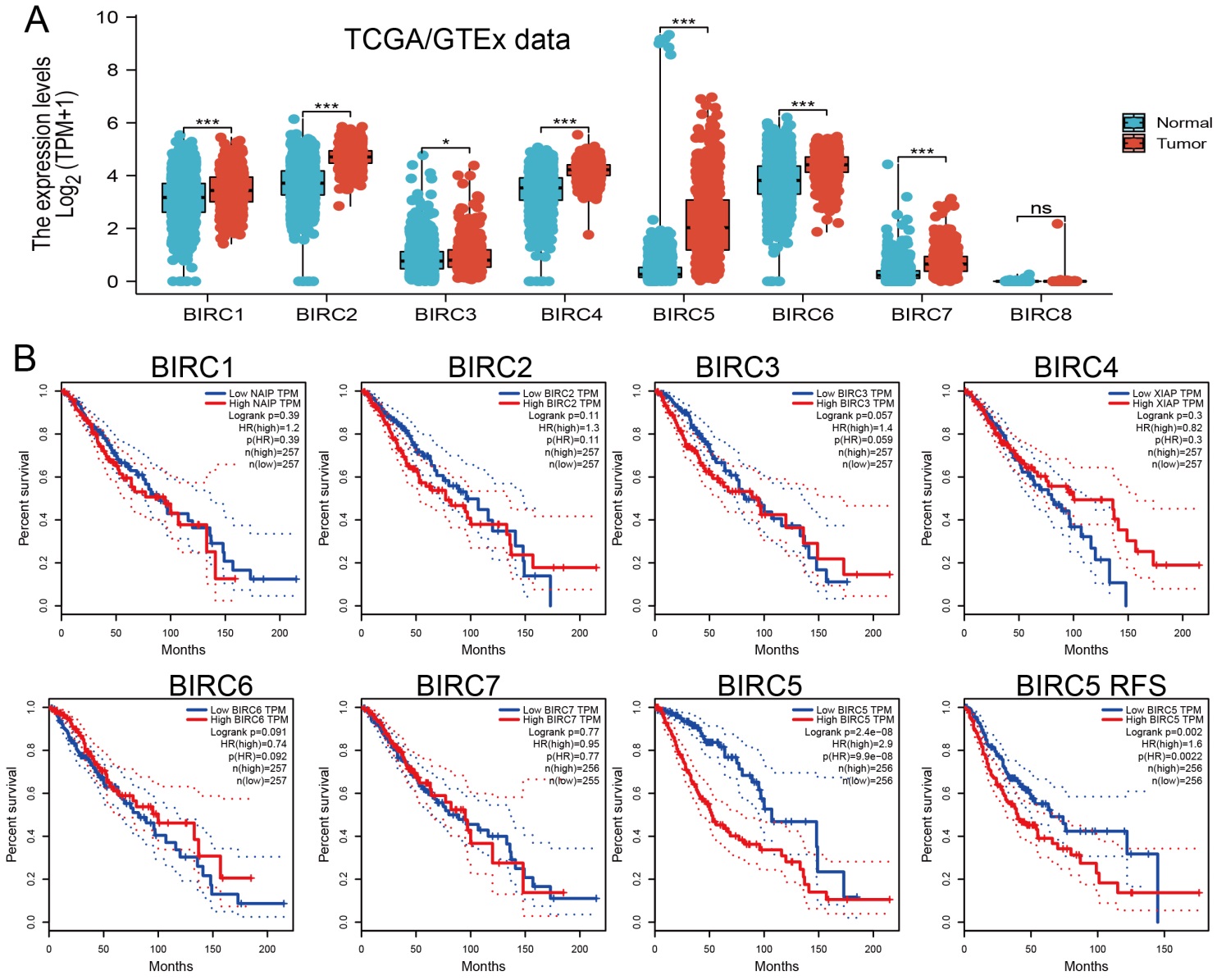
**

**Supplementary FIGURE 1 ⎜** The expression and prognosis of BIRC family gene in LGG.

**(A) The expression of the BIRC family gene in LGG was examined by TCGA/GTEx datasets. (B) The prognosis values of the BIRC family gene in LGG was examined by GEPIA database.**
